# Trials, trips, and tribulations: pathways for implementing psychedelic therapy in Ireland

**DOI:** 10.1093/ijnp/pyag028

**Published:** 2026-05-11

**Authors:** John R Kelly, Christopher Sheridan, Patricia Iusan, Lisa Coakley, Lisa Burke, Christine Brennan, Annie MacDonald, Jo-Hanna Ivers, Dominic Trepel, Garrath Tormey, Martha Finnegan, Gareth W Young, Andrew Harkin

**Affiliations:** Department of Psychiatry, School of Medicine, Trinity College Dublin, Dublin, Ireland; Trinity College Institute of Neuroscience, Trinity College Dublin, Ireland; Psychedelic Research Group, Tallaght University Hospital and Trinity College Dublin, Dublin, Ireland; Department of Psychiatry, School of Medicine, Trinity College Dublin, Dublin, Ireland; Psychedelic Research Group, Tallaght University Hospital and Trinity College Dublin, Dublin, Ireland; Department of Psychiatry, School of Medicine, Trinity College Dublin, Dublin, Ireland; Psychedelic Research Group, Tallaght University Hospital and Trinity College Dublin, Dublin, Ireland; Department of Psychiatry, School of Medicine, Trinity College Dublin, Dublin, Ireland; Psychedelic Research Group, Tallaght University Hospital and Trinity College Dublin, Dublin, Ireland; Psychedelic Research Group, Tallaght University Hospital and Trinity College Dublin, Dublin, Ireland; Psychedelic Research Group, Tallaght University Hospital and Trinity College Dublin, Dublin, Ireland; Psychedelic Research Group, Tallaght University Hospital and Trinity College Dublin, Dublin, Ireland; Psychedelic Research Group, Tallaght University Hospital and Trinity College Dublin, Dublin, Ireland; Department of Public Health and Primary Care, Trinity College Dublin, Dublin, Ireland; Psychedelic Research Group, Tallaght University Hospital and Trinity College Dublin, Dublin, Ireland; Psychedelic Research Group, Tallaght University Hospital and Trinity College Dublin, Dublin, Ireland; Department of Psychiatry, School of Medicine, Trinity College Dublin, Dublin, Ireland; Psychedelic Research Group, Tallaght University Hospital and Trinity College Dublin, Dublin, Ireland; School of Computer Science and Statistics, Trinity College Dublin, Dublin, Ireland; Trinity College Institute of Neuroscience, Trinity College Dublin, Ireland; Psychedelic Research Group, Tallaght University Hospital and Trinity College Dublin, Dublin, Ireland; School of Pharmacy & Pharmaceutical Sciences, Trinity College Dublin, Dublin, Ireland

**Keywords:** psychedelics, psilocybin, psychedelic therapy, psychedelic-assisted therapy, clinical trials

## Abstract

Classical serotonergic psychedelics, such as psilocybin, show emerging evidence of therapeutic potential across a range of mental health disorders, including depression, anxiety, and substance use disorders, with indications of transdiagnostic efficacy. While early-phase studies yielded encouraging results, recent larger-scale phase 3 trials, such as those evaluating psilocybin for treatment-resistant depression, have shown more modest effects, and further findings from ongoing trials are awaited. Despite the absence of regulatory approvals from the U.S. Food and Drug Administration and European Medicines Agency, a small but growing number of countries have permitted psychedelic therapies within regulated clinical settings. Across these divergent international approaches, the long-term trajectory and real-world impact of these therapies within public health systems remain uncertain. In anticipation of potential future approval, Ireland has an opportunity to draw on international experience and proactively plan for the integration of psychedelic therapies. Building on emerging evidence, international frameworks, and Ireland-specific policy and health system features, this paper examines the challenges of integrating psychedelic therapies into the Irish public healthcare system. These challenges span regulatory approval, Health Technology Assessment, service implementation, workforce capacity, and the evaluation of long-term patient outcomes. The aim is to inform policymakers, practitioners, and researchers about key system-level considerations.

**Summations and Perspectives**

- Serotonergic psychedelics, such as psilocybin, show potential for depression, anxiety, and substance use disorders, although formal approvals (FDA, EMA, HPRA) are absent, with controlled clinical use growing globally.
- Ireland can implement Health Services Executive (HSE)-supported pilot programs alongside a national research hub to test cost-effectiveness, track longitudinal bio-psycho-social-environmental outcomes, and advance personalized approaches, while providing oversight, training, a national registry, and easing regulatory barriers to ensure equitable and sustainable integration into the public mental health system.
- Coordinated research and inclusive governance can support evidence-based integration, build trust, and position Irish psychedelic and novel therapeutic research within European and global policy and research frameworks.

## Introduction

Mental health disorders are complex conditions that emerge from the dynamic interplay of biological vulnerabilities, early-life adversities, and a combination of psychosocial, socioeconomic, and environmental stressors. According to the Organisation for Economic Co-operation and Development (OECD) Health Profile 2023, approximately 21% of the Irish population had a mental health disorder in 2019, including 5% with depressive disorders, 7.6% with anxiety disorders, and 4.7% with substance use disorders.^1^ The estimated economic cost of mental health problems in Ireland in 2018 was €8.2 billion per year, equivalent to 3.2% of gross domestic product.^2^

Major depressive disorder (MDD) remains the leading reason for psychiatric hospital admissions in Ireland. In 2024, 22% of all admissions and 25% of first admissions were for depressive disorders.^3^ Multiple large pragmatic trials show that less than half of patients with MDD achieve remission after a single adequate course of monoamine-targeting antidepressants.^4^^,^^5^

Treatment-resistant depression (TRD) is commonly defined as failure to achieve response or remission following at least two adequate antidepressant trials during the current depressive episode.^6^ It is estimated to occur in approximately 30% of patients with MDD.^7^ For TRD, guidelines recommend a range of strategies, including combining conventional antidepressants, augmenting with lithium or atypical antipsychotics, or employing interventional or neuromodulation techniques like electroconvulsive therapy (ECT).^8–10^

Classical (serotonergic) psychedelics show increasing evidence of therapeutic potential across a range of mental health disorders (Table 1). However, the initial wave of the psychedelic renaissance with understandable enthusiasm and heightened expectations, is now encountering more modest outcomes,^92^^,^^93^ fragmented regulatory frameworks, and significant implementation challenges.^94–97^

**Table 1:** Psychedelics under clinical investigation.

Routes of administration	Onset of action	Duration of action	Metabolism/excretion	T½ elimination	Main receptor targets
Psilocybin					
Oral; IV^11^ (Psilocybin) *Molecular formula:* C_12_H_17_N_2_O_4_P *IUPAC name:* [3-[2-(dimethylamino)ethyl]-1*H*-indol-4-yl] dihydrogen phosphate	20-40 min (oral)^12^ <2 min (IV)^13^	4-6 h^12^^,^^13^	Dephosphorylation to psilocin; c. 80% hepatic glucuronidation; renal excretion, c. 20% metabolized through different pathways (including MAO, ALDH, cytochrome oxidase) and excreted through bile into stool^11^ (Psilocin) *Molecular formula:* C_12_H_16_N_2_O *IUPAC name:* 3-[2-(dimethylamino)ethyl]-1*H*-indol-4-ol	Psilocybin 160 min^14^ Psilocin 50-180 min^14^^,^^15^	5-HT_2A_^16^ 5-HT_2c_ 5-HT_1A_^17^ TrkB^18^
Key interactions			Potential indications		
• SSRIs may blunt acute effects^19^ • Buspirone ↓ visual distortion^20^ • Chlorpromazine ↓ pupil dilation/distortion^21^ • Haloperidol ↓ derealization/depersonalization, ↑ anxiety^22^ • Risperidone blunted acute effects^22^ • Ketanserin blocks subjective effects^23^			• Major depressive disorder^24^ (NCT06605105, NCT06746441, NCT05434156, NCT05385783, NCT06793397, NCT06564818) • Treatment-resistant depression^25–27^,^28^ • Comorbid depression and anxiety (NCT06820723) • Depression/anxiety in cancer^29–33^ • Depression in bipolar II disorder ^34^^,^^35^(NCT05065294) • PTSD^36^ (NCT06407635) • Post partum depression (PPD) (NCT06342310)^37^ • Comorbid depression and Parkinson’s disease^38^ • Smoking cessation^39^^,^^40^ • Cocaine use disorder (NCT02037126, NCT06102434) • Methamphetamine (NCT04982796, NCT06899594) • Obsessive-compulsive disorder (OCD)^41^ • Demoralization in AIDS survivors^42^ • Eating disorders; anorexia nervosa^43^ • Body dysmorphic disorder^44^ • Alcohol use disorder,^45^^,^^46^ NCT04718792 • Cluster headache/migraine^47–49^ (NCT04218539) • Phantom limb pain (NCT05224336) • Chronic low back pain (NCT05351541) • Comorbid chronic low back pain/MDD (NCT06355414) • Fibromyalgia^50^ (NCT05548075, NCT05068791) • Irritable bowel syndrome (NCT06206265)		
Lysergic acid diethylamide (LSD)					
Oral; sublingual; IV^51^ *Molecular formula:* C_20_H_25_N_3_O *IUPAC name:* (6a*R*,9*R*)-*N,N*-diethyl-7-methyl-4,6,6a,7,8,9-hexahydroindolo[4,3-*fg*]quinoline-9-carboxamide	30-60 min^51^	8-12 h^52^	Hepatic metabolism; urinary excretion^51^	3.6 ± 0.9 h^51^	5-HT_2A_, 5-HT_2C_, 5-HT_1A_, 5-HT_1B/D/E_, 5-HT_5A/6/7_^53^ TrkB^18^
Key interactions			Potential indications		
• SSRIs tolerated, ↓ anxiety/nausea, no autonomic/QTc changes^54^ • Chlorpromazine (IM not oral, 30 m pre-LSD) ↓ hallucinations/visual distortion. Given simultaneously or after LSD ingestion = no effect^55^ • Lithium ↑ onset/intensity^56^ • TCAs and MAOIs → earlier onset, stronger hallucinations^56^			• Depression^57^ • Anxiety^58^ • Generalized anxiety disorder^59^ • Anxiety associated with life-threatening diseases^60^ • Distress/palliative care (NCT05883540) • Alcohol use disorder^61^ • Cluster headaches (NCT03781128)		
*N,N*-Dimethyltryptamine (DMT)					
IV; inhaled; oral^62^ *Molecular formula:* C_12_H_16_N_2_O *IUPAC name:* 2-(1*H*-indol-3-yl)-*N,N*-dimethylethanamine	<10 s^63^	15-60 min^64^ 30-90 min^63^	Predominantly MAO-A deamination^62^	5-16 min^63^	5-HT_1A,1B, 1D_ 5-HT_2A,2B, 2C,_ 5-HT_5a_, 5-HT_6,_ 5-HT_7_^65^ Sigma-1^66^
Key interactions			Potential indications		
• Orally ingested DMT is normally not psychoactive unless MAO-A is inhibited. If taken with MAOI: serotonin syndrome (life-threatening), hypertensive crisis, severe interactions with many foods and medications, unpredictable psychological effects, cardiovascular complications • SSRIs: possible ↑ antidepressant effect, risk of serotonin toxicity^67^ • MAOIs ↓ subjective effects^68^ • Pindolol ↑ effects and BP^69^			• Depression^70^ NCT06094907, NCT06927076, NCT05553691 • Alcohol use disorder (NCT06070649) • Generalized anxiety disorder (NCT06051721)		
5-Methoxy-*N,N*-dimethyltryptamine (5-MeO-DMT) (atypical)					
Smoked/vaporized; intranasal; sublingual^71^ *Molecular formula:* C_13_H_18_N_2_O *IUPAC name:* 2-(5-methoxy-1*H*-indol-3-yl)-*N,N*-dimethylethanamine	Vaporization, onset 1-50 s IM, onset 1-6 min Intranasal, onset 5-7 min^72^^,^^73^	Vaporization duration 15-30 min IM duration 60 min Intranasal duration 45-60 min^72–74^	Primarily MAO-A metabolism; potentiation with MAOIs^75^	12-19 min^76^	5-HT_1A_^77^ Sigma-1^78^ 5-HT_2A_^79^
Key interactions			Potential indications		
• SSRI and MAO-A risks similar to DMT^75^ • Extreme risk with β-carbolines: agitation, hallucinations, emesis, hyperthermia, tachycardia^80^^,^^81^			• Depression/TRD^82–84^ NCT04698603, NCT05800860 • PPD^85^ • Anxiety/depression in mild cognitive impairment (NCT06812221)		
Mescaline					
Oral *Molecular formula:* C_11_H_17_NO_3_ *IUPAC name:* 2-(3,4,5-trimethoxyphenyl)ethanamine	45-90 min^86^^,^^87^ Mean peak concentration within 2 h	6-15 h^86^^,^^88^ 2.8 h (100 mg) 15 h (800 mg)^87^	Hepatic; urinary elimination^86^ Primarily renal excretion^87^	2.6-5.3 h^76^ 3.5 h^87^	5-HT_1A,_ 5-HT_2A,2B, 2C_^76^^,^^89^
Key interactions			Potential indications		
• Chlorpromazine ↓ acute anxiety in 68%^90^ • Azacyclonol attenuates subjective effects^91^			• Limited number of trials in healthy controls,^86^ NCT04227756 • No modern controlled trials in clinical conditions		

Pharmacological and clinical characteristics of classical psychedelics. Table summarizes routes of administration, onset of action, duration of action, metabolism and excretion, elimination half-life, receptor targets, key pharmacological interactions, and therapeutic indications. 5-HT, serotonin (5-hydroxytryptamine) receptor subtypes; BP, blood pressure; h, hour(s); IV, intravenous; MAO-A, monoamine oxidase A; MAOI, monoamine oxidase inhibitor; min, minute(s); OCD, obsessive-compulsive disorder; PO, oral; PTSD, post-traumatic stress disorder; SNRI, serotonin–norepinephrine reuptake inhibitor; SSRI, selective serotonin reuptake inhibitor; T½, elimination half-life; TCA, tricyclic antidepressant; TrkB, tropomyosin receptor kinase B; σ1_11, sigma-1 receptor.

To date, two phase 3 trials of psilocybin therapy for TRD have been conducted. The first compared a single 25-mg dose of psilocybin with placebo and reported a modest symptom improvement (a 3.6-point reduction on the Montgomery–Åsberg Depression Rating Scale). The other trial compared two doses of psilocybin 25 mg versus 1 mg with a similar mean treatment difference of −3.8 points.^93^ These trials will inform the U.S. Food and Drug Administration (FDA) decisions on psilocybin therapy for TRD, expected by late 2026 or early 2027.

Even without FDA and European Medicines Agency (EMA) regulatory approvals, there appears to be a growing receptivity to medical use in structured contexts.^98^^,^^99^ A select but growing number of countries and jurisdictions have permitted the use of psychedelic therapies in limited, tightly regulated clinical settings. In parallel, reflecting the expanding global infrastructure supporting psychedelic science, from academic and translational research to advocacy and policy reform, an increasing number of international organizations, foundations, professional bodies, and research consortia are actively working to integrate psychedelic therapies into public health systems. These efforts emphasize safety, equitable access beyond private care settings, the standardization of therapist training, systematic monitoring of adverse events, and robust informed consent procedures.^97^^,^^100–116^

Ireland’s small but growing and diversifying population, combined with its integrated public healthcare system, provides an opportunity to pilot and implement psychedelic therapies within existing care pathways. As part of the global, and particularly European, psychedelic research ecosystem, Ireland can both learn from international experience and contribute to a coordinated European framework, generating transferable lessons on governance, service integration, and evaluation to support a mutually informed and harmonized trajectory across countries.^116^

As psychedelic therapies advance toward regulatory consideration, this paper examines challenges to integrating psychedelic therapy within Ireland’s healthcare system, spanning regulatory approval, health technology and assessment (HTA) and reimbursement, adoption in the public mental health system, workforce capacity, and patient outcomes, with the aim of informing policymakers, practitioners, and researchers about system-level considerations.

## Navigating regulatory challenges in Ireland

Globally, approaches to the regulation of psychedelics vary both within and between countries (Table 2).^102^^,^^117–121^ Psychedelics are currently classified as Schedule 1 controlled drugs in Ireland under the Misuse of Drugs Act 1977, defined as drugs with no currently accepted medical use and a high potential for abuse. In contrast, ketamine and esketamine are Schedule 3, reflecting their medical use alongside potential for abuse.

**Table 2:** Regulatory approaches to psychedelics: international overview.

Country/region	Regulatory status	Key developments	Current challenges
United States	No formal approvals by FDA; FDA rejected MDMA-assisted therapy application for PTSD (Lykos Therapeutics, 2024)	State-level legalization of psilocybin therapy (Oregon, Colorado) FDA’s Commissioner’s National Priority Voucher Acceleration of FDA review and expansion of early access pathways, alongside increased funding for psychedelic research (2026)	Ongoing debate over federal vs. state authority; fragmented landscape Early clinical service models established; high operating costs and regulatory complexity VA exploring psilocybin integration
Canada	No formal approvals; National Special Access Program	Permits restricted medical access in exceptional cases	Fragmented regional approaches
Australia	Regulated medical use (specialist prescribing framework)	First national regulator to reclassify psilocybin and MDMA (2023), enabling prescribing access under strict conditions	Largely excluded from public reimbursement; cost limits access
New Zealand	No formal approvals	New Zealand Medicines and Medical Devices Safety Authority (Medsafe) granted individual approval to a single named psychiatrist to prescribe psilocybin for TRD under a pre-existing regulatory pathway (2025)	Limited scope; psilocybin remains an unapproved medicine
Japan	No formal approvals	Research is increasing (eg, Otsuka–Keio)	Restrictive regulatory environment
Switzerland	Restricted access (exceptional/compassionate use prescribing framework)	Licensed physicians can prescribe LSD, MDMA, psilocybin for variety of conditions	Access limited to trained physicians, outside formal regulatory approval
Germany	Restricted access (compassionate use/early access pathways for psilocybin in clinical settings)	Clinicians can prescribe psilocybin for TRD	Restricted to a single indication; early-stage implementation
Norway	No formal approvals	Public reimbursement of off-label IV ketamine for TRD within the public health system (2025), representing comparator pathway	Classical psychedelics and MDMA remain restricted to research studies
Ireland	No formal approvals; psychedelics remain Schedule 1 under Misuse of Drugs Act	Publicly funded (HRB) translational studies and clinical trial	Complex reimbursement system (NCPE, HSE, hospitals)
Czechia	Regulated medical use (specialist prescribing framework under newly enacted legislation)	Early legislative movement toward psilocybin therapy authorization without prior EMA approval	Early-stage implementation of specialist prescribing framework
EU	No formal EMA approvals	Ongoing discussion of regulatory sandbox approaches to support supervised psychedelic research; EMA PRIority MEdicines (PRIME) scheme supporting accelerated development pathways	Fragmented national implementation and absence of EU-level clinical authorization pathway for psychedelics
United Kingdom	No formal approvals	Developed research institutions and increasing links with NHS facilities and Royal College of Psychiatrists	Increasing research activity and exploratory NHS-linked pilot initiatives
South America (Brazil)	No formal approvals	Ayahuasca is legally permitted for religious use and has facilitated culturally anchored research	Limited clear pathways to integrate into mainstream mental health care and reimbursement systems

Selected representative examples of regulatory approaches to psychedelics (not exhaustive). EMA, European Medicines Agency; EU, European Union; FDA, Food and Drug Administration; HSE, Health Service Executive (Ireland); HRB, Health Research Board (Ireland); IV, intravenous; MDMA, 3,4-methylenedioxymethamphetamine; NCPE, National Centre for Pharmacoeconomics (Ireland); NHS, National Health Service (United Kingdom); PTSD, post-traumatic stress disorder; TRD, treatment-resistant depression; VA, Veterans Affairs (United States Department of Veterans Affairs).

In October 2018, the FDA granted a Breakthrough Therapy designation to COMPASS Pathways’ psilocybin therapy program for TRD, marking an early regulatory acceleration. In 2019, the FDA again awarded Breakthrough Therapy status to the Usona Institute’s psilocybin program for MDD. More recently, a novel psychedelic compound, bretisilocin, has been accepted into the EMA’s PRIME accelerated development scheme for MDD. Notably, neither the FDA nor the EMA has approved any psychedelic therapies, and in August 2024 the FDA declined Lykos Therapeutics’ application for MDMA-assisted therapy for PTSD.

Despite regulatory constraints, variable access, and therapies that remain largely outside reimbursement systems, the broader international policy landscape is becoming increasingly receptive to the therapeutic use of psychedelics within structured, approved medical contexts.^122^

### Rescheduling psychedelics in Ireland?

In 2023, Australia’s Therapeutic Goods Administration became the first national regulator in the world to reclassify psilocybin and MDMA, permitting their use in specific medical contexts.^123^ In 2025, the UK Government supported pilot exemptions to licensing requirements for Schedule 1 research in universities and hospitals.^124^ In Ireland, one potential pathway to enable clinical research and early therapeutic use is to follow the Australian model by rescheduling psychedelics to a lower schedule or creating a dedicated regulatory category, coupled with carefully designed restricted-access or compassionate-use schemes. Aligned with international precedents but tailored to the Irish context, such approaches could facilitate real-world evidence generation while maintaining appropriate oversight.

### Compassionate-use programs

Compassionate-use pathways for psychedelic therapies remain limited in Europe. Switzerland has been at the forefront of psychedelic research and psychedelic-assisted therapy (PAT), providing one of the most established frameworks for “restricted access” use and clinical delivery outside of formal marketing authorizations.^100^ Since 2014, Switzerland has permitted authorized and trained physicians across a range of medical specializations to administer LSD, MDMA, and psilocybin for selected indications, primarily depression, PTSD, and anxiety, on a case-by-case basis.^100^

In August 2025, Germany introduced a compassionate-use program for psilocybin therapy in TRD, allowing clinicians at authorized centers to seek regulatory approval for individual patients to receive psilocybin. While this represents a noteworthy step toward clinical access in Europe, psilocybin remains unapproved as a marketed medicine, and eligibility decisions are made within structured regulatory and ethical frameworks.^125^ As outlined by Liechti et al., compassionate-use/restricted-access programs offer several advantages, though decisions remain at the national level, resulting in fragmented compassionate use frameworks across Europe.^100^ Similarly, there are regional variations and divided opinions in Canada, though there is a national special access program.^126–128^

In Ireland, a compassionate-use program approach would likely be highly selective, limited to a small number of patients, with no guarantee of HSE reimbursement. Despite retaining the capacity to authorize the clinical use of certain medicines without full EMA approval, typically under restricted, exceptional, or compassionate-use provisions, the Health Products Regulatory Authority (HPRA) very rarely issues such authorizations.

Even with HPRA and EMA approval, navigating reimbursement and clinical integration remains a complex, multi-layered challenge. Ultimately, regulatory approval, whether through the HPRA, EMA, or EU regulatory sandboxes (time limited, purpose-specific frameworks designed to enable supervised testing of novel compounds outside full regulatory constraints),^129^ represents the first and necessary step, enabling HTA via National Centre for Pharmacoeconomics (NCPE) evaluation, reimbursement decisions by the HSE Drugs Group supported by the Corporate Pharmaceutical Unit (CPU), and then hospital-level approval, with each hospital following its own processes and timelines.^130–132^

### Supervised-access models

In contrast to clinician-led, medical access models, jurisdictions such as Oregon and Colorado in the United States have implemented non-clinical, facilitator-led frameworks for psilocybin that operate outside FDA approval, with a legal framework deliberately designed to avoid a strictly medical approach.^133^ Under these programs, many clients access the services with therapeutic intent, and the Oregon ballot measure itself referenced mental health goals. However, participants do not require a medical diagnosis, facilitators need not hold healthcare credentials, and Oregon’s rules explicitly forbid them from diagnosing or treating mental illness. These models have encountered cost and sustainability problems. In Oregon, approximately one-quarter of clinics have closed, citing unsustainable costs and burdensome regulatory requirements.^134^

Efforts are also underway to integrate psilocybin therapy into the Veterans Affairs System.^135^ However, opinions diverge, with some arguing that the FDA should remain the sole regulatory authority for psychedelic therapies in the United States.^133^

### Non-clinical use?

While this paper focuses specifically on clinical integration in Ireland, including service delivery models, regulatory considerations, and implementation within mental health services, it is important to acknowledge the increasing prevalence of non-clinical psychedelic use.^136–141^ Such use occurs in recreational, spiritual, or microdosing contexts and can influence public perceptions, patient expectations, and safety considerations. These developments sometimes occur alongside movements advocating for recreational use and the expansion of commercial psychedelic markets. Although these agendas occasionally overlap with clinical initiatives, conflating them risks complicating policy development, weakening ethical governance, and undermining the scientific credibility of regulated therapeutic pathways. Recognizing the distinction between non-clinical use and evidence-based clinical access underscores the need for regulated, ethical, and transparent implementation frameworks that support consistent and scientifically grounded delivery of psychedelic therapies in Ireland’s public healthcare system.

## Health technology assessment and reimbursement

In Ireland, the NCPE acts as the national HTA body for pharmaceuticals, evaluating new medicines following marketing authorization via either the EMA centralized procedure or national regulatory pathways involving the HPRA. Applicants submit reimbursement applications to the CPU of the HSE, which commissions the NCPE to conduct a rapid review of the submission. This process, typically completed within approximately 4 weeks, determines whether a full HTA is required to inform reimbursement decisions or whether a recommendation can be made based on the rapid review alone. A full HTA is generally not required when sufficient evidence exists to support a clear reimbursement decision.^142^

The HSE Drugs Group, supported by the CPU, evaluates reimbursement applications informed by HTAs conducted by the NCPE and makes recommendations to HSE leadership regarding reimbursement decisions in line with statutory criteria, including clinical need, clinical and economic evidence, budgetary implications, and available resources.^142^^,^^143^ In recent years, there has been an increasing proportion of positive or conditionally positive reimbursement determinations in Ireland.^142^

However, the overall average time from application to reimbursement in Ireland is approximately 617 days, with many medicines exceeding the 180-day target timeline referenced in EU and national reimbursement frameworks, highlighting delays in access to medicines.^144^ A 4-year framework agreement between the State and the Irish Pharmaceutical Healthcare Association aims to support more streamlined processes and improve timeliness of access.

While HTA decisions are based on the best available evidence at the time of assessment, they may be revisited as additional clinical and real-world data emerge. This is particularly relevant for psychedelic therapies, where uncertainties regarding treatment durability, service delivery models, and long-term outcomes may necessitate iterative reassessment. Across Europe, there are increasing calls for more harmonized HTA and regulatory frameworks, reflecting the need for consistent evaluation standards for emerging therapies, including psychedelics.^121^^,^^145^

### Ketamine and esketamine in Ireland: Lessons for psychedelic therapy

In Ireland, the experience with ketamine and esketamine illustrate the complexities of integrating novel therapies into clinical practice. Intranasal esketamine (Spravato) received regulatory approval from the EMA and the HPRA in 2019 as an adjunctive treatment for TRD, despite nuanced outcomes in its phase 3 trials. More recently, the FDA has expanded its approval of esketamine to include use as a monotherapy for TRD.

Esketamine received reimbursement approval from the NCPE in January 2022, following confidential price negotiations with the HSE. The NCPE initially recommended against reimbursement unless its cost-effectiveness improved, indicating concerns about clinical and economic value. The NCPE concerns echoed the United Kingdom, where the National Institute for Health and Care Excellence (NICE) did not recommend esketamine for routine use in the National Health Service (NHS) for TRD, mainly due to concerns about cost-effectiveness and uncertainties about long-term benefits.^146^^,^^147^

Even after confidential HSE price negotiations and reimbursement approval, esketamine clinical uptake in Ireland remains limited. This likely reflects a combination of factors. Partly, it may be a deliberate HTA judgment weighing cost-effectiveness and feasibility. More broadly, adoption is constrained by high treatment costs, the need for specialized infrastructure, adequately trained clinical personnel, and careful patient monitoring, all of which pose practical challenges for routine implementation.^148^ Unlike most psychedelic therapy models, ketamine treatment typically does not include preparatory or integration therapy sessions.^149–151^ These findings have direct implications for the introduction of psychedelic therapies, which may entail even higher costs depending on dosing schedule, and therefore require careful HTA evaluation and reimbursement planning.

Notwithstanding the markedly different healthcare systems, in contrast, in the United States, the esketamine market alone is currently valued at over 1 billion dollars, underpinned by a predominantly privatized healthcare system characterized by limited universal coverage and disparities in access.^152–155^ Socioeconomic factors add complexity to implementing psychedelic therapies, affecting both access and treatment response. Monitoring these determinants is essential to ensure equitable care and inform outcomes when planning national service delivery, as socio-environmental stability may modestly influence treatment effects and cost-effectiveness.

### Cost-effectiveness of psychedelic therapies: implications for the Irish Healthcare system

The cost-effectiveness of psychedelic therapies is likely to play a central role in their potential integration and long-term sustainability within public health systems. Projecting costs is complex due to multiple factors, including the price of proprietary drug formulations, the duration and frequency of preparatory and integrative therapy sessions, and the need for specialized nurse time.^156^

Estimates of the per-patient cost of psilocybin therapy vary considerably. Average estimated costs are around €10 000 per patient,^157^ though depending on the therapeutic model and pricing, total costs may approach €20 000, comparable to a course of esketamine treatment. This highlights the importance of the durability of therapeutic effects when evaluating overall value and cost-effectiveness.^29^^,^^158^ A recent cost-effectiveness analysis in the United States found that to be considered economically favorable compared with available treatments for TRD, psilocybin therapy would need to cost $5000 or less.^159^

In Switzerland, the full cost of a single day of psychedelic therapy is estimated at €3000-€4000, though patients are typically charged between €800 and €2000. This treatment is currently not fully reimbursed by mandatory health insurance and is usually self-funded.^100^ Typically, patients undergo 2 to 4 sessions over a 12-month period. The cost of the psychedelic substance itself ranges from €100 to €450.

In Australia, the cost of psychedelic therapy is approximately €8000-€12 000 per patient and is not covered by health insurance, making it financially inaccessible for many individuals.

Ireland’s cost-effectiveness threshold of approximately €45 000 per quality-adjusted life year (QALY, a metric combining quality and quantity of life) is broadly aligned with thresholds used in the United Kingdom and Canada.^160^^,^^161^ As Muthukumaraswamy et al. note for New Zealand,^162^ which has a population comparable to Ireland, if 2% of the population (approximately 100 000 people) were to seek psychedelic therapy at a cost of $15 000 per person, total expenditure would approach $1.5 billion, exceeding New Zealand’s entire annual medicines budget.

By comparison, assuming a conservative 5% prevalence of moderate-to-severe depression in Ireland^1^ (approximately 255 000 people) and that 30% of these individuals meet criteria for TRD, approximately 76 500 people may have TRD. Providing psychedelic therapy to this population at a cost of €10 000 per patient would amount to €765 million, or roughly 20% of Ireland’s total medicines budget. In practice, initial uptake would likely be substantially lower, with treatment focused on selected patients through phased HSE pilot programs, requiring careful prioritization, planning, and potentially innovative solutions prior to expanding to later phases of rollout.

## Navigating Ireland’s leverage and constraints in psychedelic innovation

Pharmaceutical innovation often involves trade-offs, with innovation and equity frequently in tension.^148^ These tensions are particularly acute in publicly funded health systems like Ireland’s, where broader concerns about equitable access and cost-effectiveness influence policy decisions.

While classical psychedelics are long off-patent, companies increasingly seek intellectual property protection for novel formulations, delivery methods, and treatment protocols.^163–166^ At the same time, such protections are often viewed as necessary to incentivize investment, as without them clinical trials may not be completed and the development of improved compounds could be limited.

Ireland’s EU membership, established pharmaceutical manufacturing base, and potential as a European early-mover in coordinated psychedelic regulation could provide leverage beyond what raw market size might suggest. However, despite the presence of many global pharmaceutical companies conducting research and manufacturing operations in Ireland, the country has relatively few significant indigenous pharmaceutical companies and conducts fewer clinical trials per capita than peer European countries. For instance, Denmark conducts nearly three times as many industry-sponsored trials.^167^ Within the psychedelic sector, only a handful of Irish-founded companies, such as GH Research and Alvarius Pharmaceuticals, are currently active.

Taken together, these factors raise important questions about the extent to which Ireland can meaningfully influence the terms under which psychedelic therapies and novel therapeutics enter its market.

## Adoption of psychedelic therapy within the public health service in Ireland

If approved, pilot sites or institutions should proactively identify sustainable implementation approaches to support effective integration. Cost-effectiveness within the Irish public health system could be enhanced by embedding psychedelic therapy into pre-existing community mental health infrastructure, staffing, and established governance structures. Approaches such as group-based delivery models and/or online or video-based sessions for preparation and integration sessions, particularly for those in remote areas, where appropriate, could further optimize resource use.^168–171^

### Integrating psychedelic therapy within interventional Psychiatry?

As new therapeutic modalities emerge, questions arise regarding the most appropriate clinical and organizational frameworks for their delivery. Interventional psychiatry is a subspecialty focused on neuromodulatory treatments, such as ECT, repetitive transcranial magnetic stimulation (rTMS), and ketamine/esketamine, as well as emerging novel pharmacological agents, including psychedelic compounds.^172^ This field primarily addresses treatment-resistant conditions through targeted, often procedure-based interventions beyond standard psychopharmacology.

In Ireland’s public mental health system, interventional psychiatry is largely restricted to ECT, with access integrated across all local services.^173–178^ Access to other emerging modalities such as rTMS and most esketamine/ketamine treatments are largely restricted to private services or clinical trials, or a very limited number of select settings in public mental health services in Ireland.^179–182^

Structurally, incorporating psychedelic therapies into emerging interventional psychiatry services may represent a logical progression of current models. The clinical populations served by psychedelic therapies often differ from those typically treated with other interventional modalities such as ECT, which is more commonly employed in older adults with more severe presentations, psychotic disorders, or marked psychomotor disturbance.^183^ Conversely, psychedelic therapies are contraindicated in psychosis and may have more modest effects in older adults,^184^ whereas certain complex personality difficulties, particularly borderline personality traits may moderate treatment response in both ECT and psychedelic therapies.^185^^,^^186^

Currently in Ireland, apart from ECT, cohesive interventional psychiatry pathways are not fully established. ECT is monitored through a national mandatory registry, and the anticipated approval of psychedelic therapies provides an opportunity to extend this approach. A mandatory registry for psychedelic therapies could systematically collect data across sites, facilitate monitoring of outcomes, inform service design, guide policy, track long-term effects, and support transparency and public trust.^187^ Pragmatically aligning psychedelic therapies with existing interventional psychiatry services, rather than creating parallel structures, would allow Ireland to build on established governance mechanisms, such as registries and multidisciplinary teams, while avoiding duplication of resources. This approach would not only broaden interventional psychiatry beyond ECT but also ensure that proposed infrastructural investments could support a wider range of novel therapeutics, rather than being contingent on a single class of compounds.

### Workforce capacity and training

#### Psychedelic therapy: training and regulatory considerations in the Irish context

While therapy appears to be synergistic with psychedelics, particularly through the quality of the therapeutic relationship, the optimal number, type, and structure of preparatory and integration sessions remain to be established, and the qualifications required for therapists delivering such interventions are not yet clearly defined.^188–199^ Notably, some trials do not provide formal or manualized therapy, highlighting ongoing variability in treatment approaches and the absence of consensus regarding optimal therapeutic frameworks.^82^

Regulatory authorities do not regulate psychotherapy itself, but are increasingly acknowledging the importance of therapeutic context in psychedelic clinical trials. The EMA’s updated Guideline on the Clinical Investigation of Medicinal Products in the Treatment of Depression includes a dedicated section on psychedelics that recognizes the methodological challenges of psychedelic therapy, including the need to define and document psychological support and preparatory/integration components within trial protocols.^98^ The guideline notes that the type, duration, and frequency of psychological interventions and associated training should be standardized as much as possible, despite ethnic and cultural differences, and that plans for translating therapist training from trial to clinical practice must be addressed.^98^ Additionally, trials must demonstrate that observed therapeutic effects are not solely attributable to the psychological intervention.^98^

Similarly, the FDA’s draft guidance on psychedelic drugs outlines considerations for how psychological support should be addressed in trial design and interpretation but does not require specific therapy modalities or training transfer plans in regulatory submissions.^99^

As of 2026, counselors and psychotherapists in Ireland are not statutorily regulated under the Health and Social Care Professionals Act 2005. Instead, self-regulation predominates through professional bodies such as the Irish Association for Counselling and Psychotherapy and the Irish Council for Psychotherapy, which establish professional standards and oversee member accreditation. In contrast, professions such as clinical psychology are statutorily regulated, highlighting a regulatory asymmetry that may have implications for the delivery of psychedelic therapy. Plans to regulate psychotherapists and counselors in Ireland are at an advanced stage, moving toward statutory regulation under CORU (Ireland’s multi-profession health regulator). However, the current absence of statutory regulation represents a potential gap in defining who is authorized to deliver the psychological component of such interventions, under what professional authority, and within which regulatory framework.

An initial pathway for consideration in Ireland could involve the development of an accredited postgraduate program in psychedelic therapy, coupled with a credentialing pathway for practitioners that bridges existing professional boundaries. Aligning such training within an academic institution and with HSE service delivery would allow Ireland to establish cohesive, accessible, standardized, evidence-based training pathways, and an integrated training framework, informed by lessons from other jurisdictions.^95^^,^^200^^,^^201^

Recently, the Royal College of Psychiatrists published guidance on Psychotherapy Assisted by Psychedelic and Related Substances,^202^ which could serve as a starting reference for the Irish College of Psychiatrists in developing national guidance and establishing training and learning outcomes for emerging therapeutics. Within the Irish psychiatry training scheme, the inclusion of learning outcomes related to the adoption of novel therapeutics could also be considered.

#### Public participation in the development of psychedelic clinics within the HSE

Ireland’s public health policy framework, including Sharing the Vision, places a strong emphasis on equitable access and service user involvement. As public support for psychedelic therapies continues to grow,^203^ there is an opportunity to ensure that any future HSE-delivered model is shaped by the perspectives of service users, carers, and communities. Realizing this will require the formation of a broad coalition, including clinicians (such as members of Irish Doctors for Psychedelic-Assisted Therapy), researchers, funders, policymakers, and members of the public, to collaboratively develop an ethical, evidence-based, and sustainable model for psychedelic therapy in Ireland.^204^

Recent surveys in Ireland indicate growing support for psychedelic therapies, with approximately 60% of service users supporting psilocybin therapy as a medical treatment.^203^ There is also strong conditional support among psychiatrists in Ireland. In a self-selected survey sample, the overwhelming majority (86%) indicated they would be willing to refer patients for psilocybin therapy if it were clinically indicated and licensed.^205^

These studies also highlight important concerns. The primary concern reported by psychiatrists was the currently modest evidence base, highlighting the need for further data to guide clinical and policy decisions. Durability of therapeutic effects and comparative effectiveness relative to existing evidence-based and less resource-intensive interventions were also highlighted as concerns.^205^

A co-produced approach to psychedelic therapy could help foster transparency and trust, align expectations with the evolving evidence base, and clarify eligibility criteria and outcomes that are most relevant to service users and their families. This reinforces the need for standardized, evidence-based care pathways that are not only clinically effective but also patient-centered and ethically robust, including continuity of care and long-term follow-up, key considerations for any future publicly delivered psychedelic service.^206^^,^^207^ A formal co-creation model could support this process through the establishment of a national advisory group comprising clinicians, researchers, patients, and advocacy organizations. By embedding participatory governance within a specialized psychedelic service, Ireland could set a precedent for transparency and accountability, ensuring that enthusiasm for psychedelic therapy does not outpace evidence or equity.^208^

#### Establishing a psychedelic and novel therapeutics working group within the college of psychiatrists of Ireland

One potential pathway for advancing psychedelic therapy in Ireland would be to establish a multi-stakeholder psychedelic and novel therapeutics working group within the College of Psychiatrists of Ireland. This group could provide a structured platform for clinical collaboration, research, policy development, and the formation of research networks. In Irish psychiatry, where formal frameworks for novel treatments are still evolving, the creation of a national, multidisciplinary working group could represent a pragmatic step forward. By convening expertise early, even prior to formal regulatory approval, such a group could help shape national protocols and guidelines, inform training curricula and data collection efforts, foster collaboration with national and international research networks, and advise on the phased integration of psychedelic therapies into Ireland’s public mental health system.

### Evaluating real-world impact of psychedelic therapy in Ireland

#### Roadmap to a specialized psychedelic therapy clinic in Ireland

In Ireland, the HSE is responsible for implementing government policy and delivering health and social services across hospital and community settings. The development of a dedicated pilot specialist clinic within the public mental health service may be considered in the event that psychedelic therapies receive regulatory approval. Its feasibility will depend on addressing key challenges related to clinical governance, workforce capacity, and service implementation.

#### Positioning psychedelic therapies within the treatment algorithms

The integration of psychedelic therapies into clinical practice raises key questions about their positioning within existing treatment algorithms and how they may align with or diverge from established mental health pathways in Ireland. From a pathway perspective, Ireland could begin by embedding psychedelic therapies into existing referral systems for TRD, mirroring the current positioning of esketamine, but with a potentially stronger emphasis on integrated psychological support.

Although these therapies are often positioned for refractory or treatment-resistant conditions, psychedelic interventions may ultimately prove more suitable for individuals with less severe or earlier-stage presentations. Indeed, they may function either as primary treatment modalities or as adjunctive interventions.

Should these therapies receive regulatory approval, prescribing practices are likely to expand beyond the narrow criteria typical of clinical trials, enabling access for more diverse patient populations. Realizing their full potential will require flexibility within clinical and regulatory frameworks, including adaptable eligibility criteria to promote equitable access and enhance real-world applicability, as their use will likely extend beyond TRD to a broader spectrum of disorders (Table 1). However, at this point and given the lack of clear superiority over SSRIs^209^^,^^210^ and the costs involved for the public health system, it would be prudent, at least initially, to ensure that two first-line pharmacological treatments have been trialed before proceeding along the psychedelic therapy pathway for TRD.

#### Service delivery models

**Figure 1:**
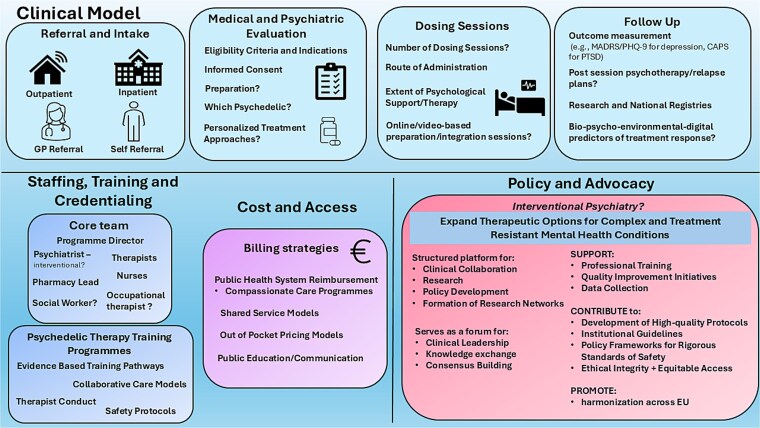
Clinical model and key considerations for psychedelic therapy. Key considerations include referrals, assessment and screening, multidisciplinary staffing, training, follow-up, cost and access considerations, and policy. MADRS, Montgomery–Åsberg Depression Rating Scale; PHQ-9, Patient Health Questionnaire-9; CAPS-5, Clinician-Administered PTSD Scale for DSM-5).

It is not yet clear what model of service delivery would be most appropriate for psychedelic therapy in Ireland (Figure 1). One potential approach is a hub-and-spoke model, similar to the delivery of ECT services in Ireland and consistent with emerging interventional psychiatry service structures, whereby Community Mental Health Teams (CMHTs) conduct initial assessments and determine suitability for treatment. Patients deemed appropriate could then be referred to specialist centers with the clinical expertise, infrastructure, and governance capacity required to deliver psychedelic therapy. These centers could also provide preparation and integration sessions, including remote delivery where appropriate, while maintaining close coordination with the referring CMHT. Further research is required to define referral criteria and determine the optimal geographic distribution of specialist centers to ensure equitable access, particularly for underserved and marginalized populations.

An alternative model would involve CMHTs directly delivering preparation and integration components. However, given existing workload pressures and resource constraints, this approach may be challenging to implement effectively in the initial rollout phase.

In the current system, general practitioners (GPs) would likely continue to refer patients to CMHTs, who would assess suitability and coordinate access to specialist centers while maintaining ongoing care. In the longer term, and contingent on appropriate training, governance, and resource development, GPs may take on a more active role in screening patients using structured criteria and facilitating referral pathways. However, these remain operational questions requiring further evaluation.

## Toward a national psychedelic research program in Ireland?

In light of the service delivery, governance, and regulatory considerations outlined above, a National Psychedelic Research Programme in Ireland could provide a structured framework to generate evidence, refine clinical and service delivery models, inform policy, and support the phased integration of psychedelic therapies and other emerging novel therapeutics into clinical practice. Reflecting Ireland’s growing alignment with international developments,^100^^,^^211–213^ a dedicated psychedelic research group has been established at Trinity College Dublin, building on the established work of the Depression Neurobiology Research Group.^176^^,^^179^^,^^180^^,^^214–220^ This foundation positions Ireland to formalize a coordinated national program that supports an evidence-led approach to translating emerging therapies into clinical practice.

**Figure 2:**
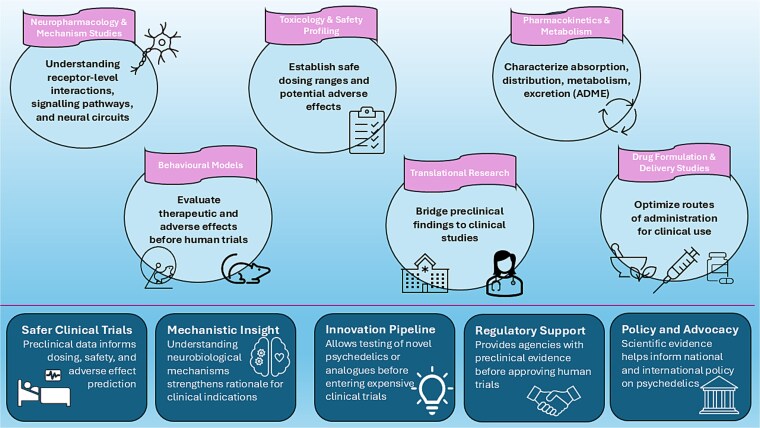
Preclinical to clinical translational model for psychedelic therapy. Illustrating mechanistic studies, animal models, and pharmacology and application to clinical practice.

A coordinated national program would also help address key evidence gaps,^221^^,^^222^ including the integration of biological, psychological, and socio-environmental mechanisms linking preclinical laboratories^223–232^ with clinical trial units and enabling iterative feedback in which preclinical findings inform trial design, while clinical outcomes guide further experimental work (Figure 2). This could advance personalized approaches by identifying patient subgroups most likely to benefit, including those who may preferentially respond to psychedelic therapies relative to other interventions or according to differences in pharmacological profiles (eg, duration of action), and by optimizing the durability of therapeutic effects.^25–27^^,^^82^^,^^209^^,^^233^^-^^247^

Practical questions regarding the interaction of psychedelic therapies with commonly prescribed medications,^248^ as well as the structure and intensity of accompanying psychological support, remain particularly important for safe and effective implementation, in addition to addressing key operational and service delivery challenges.

Over time, if tangible benefits accrue, a national psychedelic research program could evolve into a broader interdisciplinary Drug Science Centre, embedding psychedelic research alongside other emerging and novel therapeutic interventions and fostering translational, systems-oriented approaches. A national center in Ireland could be structured around interconnected pillars: (1) a research hub advancing mechanistic and translational science; (2) a clinical trials unit conducting early and late phase studies; (3) an implementation and health-services research arm evaluating real-world effectiveness, cost-effectiveness, and integration with care pathways, including coordination with HSE CMHTs; and (4) an education and training platform to build multidisciplinary workforce capacity. This integrated model would create a continuous pipeline from discovery to implementation, ensuring that evidence efficiently informs Irish clinical practice and policy for psychedelic therapies as well as other emerging novel therapeutics.

## Summary, conclusions, and future directions

As psychedelic therapies approach potential regulatory approval, their long-term trajectory and real-world impact on the Irish public mental health system remain uncertain, requiring careful alignment of regulatory frameworks, clinical processes, and logistical support. Informed by international experience, Ireland has the opportunity to shape their integration deliberately rather than reactively. This paper identifies key challenges and proposes concrete pathways forward.

Future efforts should prioritize independent research, national infrastructure, and evidence-based regulatory frameworks, including consideration of rescheduling psychedelics to a lower schedule in appropriate clinical contexts.

Without deliberate strategies to integrate delivery within HSE infrastructure, supported by training pathways, sustainable reimbursement models, and outcome tracking, psychedelic therapies and other novel treatments risk remaining confined, with limited availability in routine public healthcare in Ireland.

An HSE-supported pilot program, organized through a phased hub-and-spoke model, could test feasibility, cost-effectiveness, and referral pathways within existing community mental health infrastructure. Embedding psychedelic therapies within interventional psychiatry may help establish governance structures, national registries, and training pathways, anchoring delivery within existing clinical frameworks, although practical feasibility remains to be determined.

In parallel, a national psychedelic research hub could generate real-world evidence, support precision patient stratification and augmentation strategies, integrate digital tools, and track bio-psycho-social-environmental outcomes, ensuring that Irish data inform national policy.^249^ By linking mechanistic science, clinical delivery, and health-system integration, such a hub could serve as a single interface for researchers, regulators, and policymakers, provide training infrastructure, and maintain a national registry for safety and outcomes, with broader applicability to other emerging novel therapeutics. This coordinated approach would reduce duplication, optimize public resource use, and facilitate combined efficacy and health system studies, producing data to inform national decision-makers while also contributing to harmonized European research and policy initiatives.

By committing to pragmatic pilots, coordinated research, and inclusive governance early on, Ireland can help address current implementation gaps and operational uncertainties, ensuring that psychedelic therapies are integrated into a cohesive, equitable, and sustainable public health framework. This approach fosters trust, supports responsible stewardship, and positions Ireland to contribute meaningfully to European system-oriented psychedelic research.

## Limitations

This narrative review focuses on potential pathways for implementing psychedelic therapy within the regulated healthcare system in Ireland. Our tables highlight selected representative examples and are not intended to be exhaustive. We apologize to authors and research groups whose work we were unable to cite due to space constraints, and we further acknowledge that the scope of this work is largely limited to Western psychedelic research groups.

## Data Availability

No new data were generated or analyzed in this review.
